# ω‐Imidazolyl‐alkyl derivatives as new preclinical drug candidates for treating non‐alcoholic steatohepatitis

**DOI:** 10.14814/phy2.14795

**Published:** 2021-03-26

**Authors:** Torsten Diesinger, Alfred Lautwein, Vyacheslav Buko, Elena Belonovskaya, Oksana Lukivskaya, Elena Naruta, Siarhei Kirko, Viktor Andreev, Radovan Dvorsky, Dominik Buckert, Sebastian Bergler, Christian Renz, Dieter Müller‐Enoch, Thomas Wirth, Thomas Haehner

**Affiliations:** ^1^ Chair of Biochemistry and Molecular Medicine Faculty of Health/School of Medicine Witten/Herdecke University Witten Germany; ^2^ Department of Internal Medicine Neu‐Ulm Hospital Neu‐Ulm Germany; ^3^ Institute of Physiological Chemistry University of Ulm Ulm Germany; ^4^ Division of Biochemical Pharmacology Institute of Biochemistry of Biologically Active Compounds National Academy of Sciences Bulvar Leninskogo Komsomola Grodno Belarus; ^5^ Department of Biotechnology University of Medical Sciences Białystok Poland; ^6^ Department of Medical Biology and Genetics Grodno State Medical University Grodno Belarus; ^7^ Institute of Biochemistry and Molecular Biology II Medical Faculty of the Heinrich Heine University Düsseldorf Düsseldorf Germany; ^8^ Max Planck Institute of Molecular Physiology Dortmund Germany; ^9^ Department of Internal Medicine II University Hospital Ulm Ulm Germany

**Keywords:** cytochrome P450 2E1, non‐alcoholic steatohepatitis, reactive oxygen species

## Abstract

Cytochrome P450 2E1 (CYP2E1)‐associated reactive oxygen species production plays an important role in the development and progression of inflammatory liver diseases such as alcoholic steatohepatitis. We developed two new inhibitors for this isoenzyme, namely 12‐imidazolyl‐1‐dodecanol (I‐ol) and 1‐imidazolyldodecane (I‐an), and aimed to test their effects on non‐alcoholic steatohepatitis (NASH). The fat‐rich and CYP2E1 inducing Lieber‐DeCarli diet was administered over 16 weeks of the experimental period to induce the disease in a rat model, and the experimental substances were administered simultaneously over the last four weeks. The high‐fat diet (HFD) pathologically altered the balance of reactive oxygen species and raised the activities of the liver enzymes alanine aminotransferase (ALT), aspartate aminotransferase (AST), alkaline phosphatase (AP) and γ‐glutamyl‐transferase (γ‐GT); lowered the level of adiponectine and raised the one of tumor necrosis factor (TNF)‐α; increased the hepatic triglyceride and phospholipid content and diminished the serum HDL cholesterol concentration. Together with the histological findings, we concluded that the diet led to the development of NASH. I‐ol and, to a lesser extent, I‐an shifted the pathological values toward the normal range, despite the continued administration of the noxious agent (HFD). The hepatoprotective drug ursodeoxycholic acid (UDCA), which is used off‐label in clinical practice, showed a lower effectiveness overall. I‐ol, in particular, showed extremely good tolerability during the acute toxicity study in rats. Therefore, cytochrome P450 2E1 may be considered a suitable drug target, with I‐ol and I‐an being promising drug candidates for the treatment of NASH.

## Graphical abstract

We developed two new inhibitors of Cytochrome P450 2E1 (CYP2E1), namely 12‐imidazolyl‐1‐dodecanol (I‐ol) and 1‐imidazolyldodecane (I‐an), to reduce the reactive oxygen species (ROS) production and aimed to test their effects on non‐alcoholic steatohepatitis (NASH). Our findings suggest that CYP2E1 may be considered a suitable drug target, with I‐ol and I‐an being promising drug candidates for the treatment of NASH.

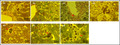

## 1 INTRODUCTION

The prevalence of non‐alcoholic fatty liver disease (NAFLD) has been estimated to be 20–30% in Western countries. A meta‐analysis of epidemiological data published recently estimated the global presence of NASH to be 59% of biopsy‐confirmed patients with NAFLD and collectively implied an association with metabolic syndrome (Younossi et al., 2016). NASH can be a terminal liver disease and belongs to the spectrum of NAFLD, which also includes liver steatosis, liver fibrosis, liver cirrhosis, and hepatocellular carcinoma (Bessone et al., 2019; Younossi et al., 2016).

Both NASH and alcoholic steatohepatitis (ASH) are defined by similar outcomes based on histological assessments of liver biopsies conducted on patients (Ludwig et al., 1980). Approximately a decade ago, NAFLD/NASH was first diagnosed almost exclusively by abdominal sonography and the determination of liver enzyme activity in the serum; the most prominent of these enzymes were alanine aminotransferase (ALT) and aspartate aminotransferase (AST) (Vernon et al., 2011). Currently, computed tomography (CT) and magnetic resonance imaging (MRI) examinations, particularly magnetic resonance elastography (MRE), are used to characterize the different stages of fibrosis (Ahmed, 2015; Drescher et al., 2019). A large panel of markers for the early detection of NAFLD has already been discussed previously (Sharma et al., 2017). However, to date, liver biopsy has been the only reliable proof for the diagnosis of NASH, provided alcohol, viral infection, toxins, or an autoimmune background is excluded.

The formation and progression of NFALD is largely determined by fat concentration in the liver and serum, but the predominant importance of free fatty acids (Yamaguchi et al., 2007) or triglycerides (Engin, 2017) is debatable. Without doubt, CYP2E1 as a fatty acid metabolizing enzyme plays an important role in the pathogenesis of NASH, wherein hepatic mRNA level (Aljomah et al., 2015), protein expression (Weltman et al., 1998) and enzymatic activity (Chalasani et al., 2003) are increased in this pathogenesis. Analogous to the pathogenesis of ASH, a causal link between NASH and oxidative stress by reactive oxygen species (ROS) can be established by CYP2E1 (Robertson et al., 2001). In addition, CYP2E1 has been recently reported to be linked to IR (insulin resistance) via the anti‐apoptotic protein Bax inhibitor‐1 (BI‐1) (Lee et al., 2016)). The latter protein plays an important role in the regulation of CYP2E1, thereby regulating ER/ROS stress (Kim et al., 2009).

Currently, there is no target‐based therapy for NAFLD, independent of the stage of the disease (Puri, 2007). Lifestyle interventions are recommended at an early stage. End‐stage liver disease is an indication for liver transplantation; however, a definitive cure is only possible in some patients. Almost one‐third of the patients who undergo liver transplantation for NASH experience disease recurrence in the transplanted liver (Bhagat et al., 2009).

**FIGURE 1:**
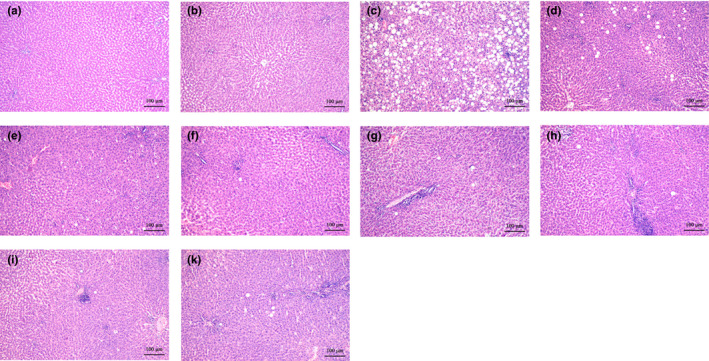
Representative histological images of liver sections from the ASH‐rat study: (a) Control, (b) isocaloric +solvent, (c) EtOH +solvent, (d) I‐ol (0.4 mg/kg b.w.) + EtOH, (e) I‐ol (4 mg/kg b.w.) + EtOH, (f) I‐ol (40 mg/kg b.w.) + EtOH, (g) I‐an (0.4 mg/kg b.w.) + EtOH, (h) I‐an (4 mg/kg b.w.) + EtOH, (i) I‐an (40 mg/kg b.w.) + EtOH, and (k) UDCA (40 mg/kg b.w.) + EtOH. Nine sections of each liver were prepared, with six or eight animals per group. All sections were stained with H. E. (original magnification: 100×; each scale bar indi‐cates 100 μm). EtOH =5% (m/m) ethanol in drinking water. Isocaloric =addition of maltodextrin to compensate for the pure caloric effect of ethanol

The two drug candidates I‐ol and I‐an were able to provide proof‐of‐concept of ASH in a rat model (Figure 1) (Diesinger et al., 2020). The central importance of CYP2E1 also in NASH should be investigated in a further proof‐of‐concept study. This study attempted to answer the following questions: (1) Can the suppression of ROS stress that had not been addressed in the treatment of NAFLD or NASH to date lead to an improvement in clinical parameters? (2) Can the CYP2E1 inhibitors successfully tested in alcoholic steatohepatitis (Table 1) cause this therapeutic effect? Considering the above‐mentioned association across CYP2E1, ROS, and NASH, we aimed, in this study, to test these inhibitors in a rat model of HFD‐induced by NASH. (3) Do the two drug candidates show a therapeutic superiority over the naturally occurring bile acid UDCA, which was used as a reference drug? (4) The acute toxicity of these inhibitors has been tested in rats, according to the ICH guidelines. Is it low enough to advance their preclinical development?

**TABLE 1:** All imidazole ring system‐containing compounds tested in vitro: The chemical compounds shown are the inhibitors of CYP2E1 that have been investigated in the context of rational drug development for the therapy of ASH. The first three inhibitors are I‐ol, I‐an, and I‐phosphocholine, which were investigated in this study

	Compound	Structure	Formula	Molecular weight
ω‐imidazolyl‐alkyl derivative	I‐ol	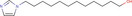	C14H27N2O	239.38
ω‐imidazolyl‐alkyl derivative	I‐an	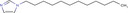	C15H28 N2	236.39
ω‐imidazolyl‐alkyl derivative	I‐phosphocholine	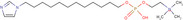	C20H41N3O4P	418.53
ω‐imidazolyl‐alkyl derivative	10‐Imidazolyl‐decanol	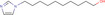	C13H24N2O	224.34
ω‐imidazolyl‐alkyl derivative	9‐Imidazolyl‐nonanol	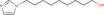	C12H22N2O	210.32
ω‐imidazolyl‐alkyl derivative	7‐Imidazolyl‐heptanol	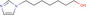	C10H18N2O	182.26
Natural library compound	STOCK1 N−69212	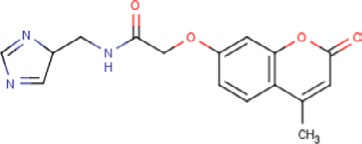	C17H17N3O4	327.34
Natural library compound	STOCK1 N−29633	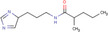	C12H21N3O	223.32
Natural library compound	STOCK1 N−57408	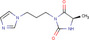	C10H14N4O2	222.24
synthetic library compound	STOCK6S−53360	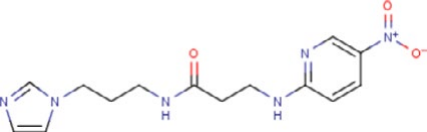	C14H18N6O3	318.33
synthetic library compound	STOCK1S−00578	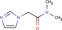	C7H11N3O	153.18
synthetic library compound	STOCK4S−67828	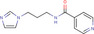	C12H14N4O	230.27
synthetic library compound	STOCK5S−82936	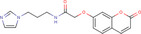	C17H17N3O4	327.34
synthetic library compound	STOCK6S−46082	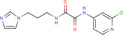	C13H14ClN5O2	307.74
synthetic library compound	STOCK4S−27365	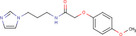	C15H19N3O3	289.33
synthetic library compound	STOCK3S−47400	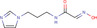	C8H12N4O2	196.21

## 2 MATERIALS AND METHODS

### 2.1 Animals, diets, and treatment

Chemical synthesis of the active agents I‐ol and I‐an was performed at the University of Ulm. UDCA had been acquired from Prodotti Chimici e Alimentari S.p.A., Hydroxypropylmethylcellulose (Hypromellose, HPMC) from Shin‐Etsu Chemical Co., Ltd. All other chemicals were of highest chemical and biological purity grades.

### 2.2 Establishment of NASH in rat model

The care and use of experimental animals and the procedures performed using them conformed to the institutional guidelines, national and international laws, and the guidelines set as per Directive 2010/63/EU of the European Parliament and of the Council of 22.10.2010 on the protection of animals used for scientific purposes. The local ethics committee for animal experiments in Grodno and the Ethics Committee of the National Academy of Sciences, Belarus approved the study.

Studies were done using Wistar rats of female sex by the Institute of Pharmacology and Biochemistry at Minsk. The animals were 80–90 days old and weighed 180–200 g. They had free access to a liquid Lieber‐DeCarli (LDC) diet (Lieber et al., 2004) and were housed under standard conditions meaning a 12‐h light/dark cycle at 22 ± 2°C and 55 ± 5% humidity.

**FIGURE 2:**
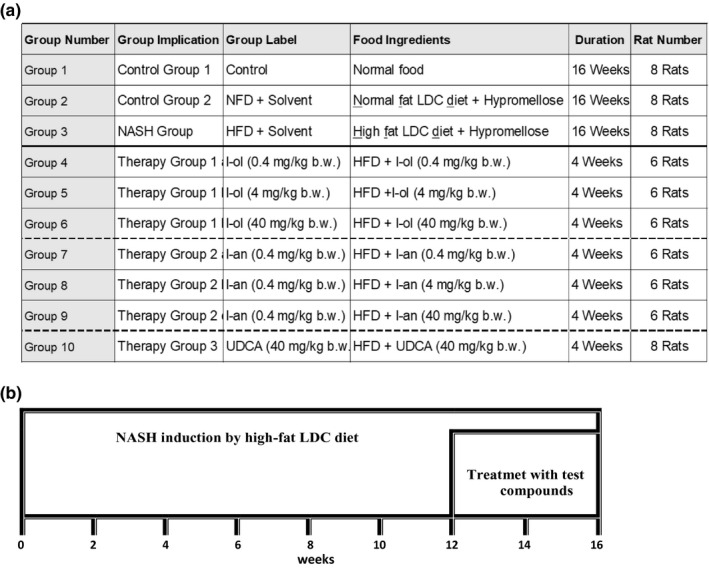
Scheme of animal experiments: (a) Group differences: Rats were divided into two control groups, one disease group, and seven treatment groups. The disease group and treatment groups received an HFD, with 4.9% (m/m) corn oil. (b) Time frame: The animal study lasted 16 weeks. While all diets were administered over the entire period of study, the corresponding test compounds were only administered in the last four weeks. The test compounds and hypromellose were administered daily via oral gavage

The rats were divided into 10 groups based on the diets and experimental substances administered (Figure 2).

Group 1 was designated as the first control group; it was fed a normal standard solid diet. Group 2, as the second control group, was fed a standard amount of fat as a part of the liquid “physiological,” non‐high fat LDC diet. All other groups received the liquid ‘disease‐causing’ LDC diet (Table 2), including a high content of unsaturated fatty acids.

**TABLE 2:** Composition of the liquid Lieber‐DeCarli (LDC) diet: The composition of the NFD and HFD was obtained from the study by Lieber et al., 2004. [Lieber CS, Leo MA, Mak KM, Xu Y, Cao Q, Ren C, et al. Model of nonalcoholic steatohepatitis. Am J Clin Nutr. 2004;79(3): 502–9]

Compounds	LDC diet with normal fat content		NASH diet	
	(g)	(%)	(g)	(%)
Casein	41.4	4.1	41.4	4.1
L‐Cysteine	0.5	0.1	0.5	0.1
DL‐Methionine	0.3	0.0	0.3	0.0
Maltodextrin	25.6	2.6	25.6	2.6
Cellulose	10.0	1.0	10.0	1.0
Minerals‐VM	8.8	0.9	8.8	0.9
Vitamins‐VM	2.5	0.3	2.5	0.3
Choline chloride^a^	0.5	0.1	0.5	0.1
Guar gum^b^	3.0	0.3	3.0	0.3
**Corn oil**	8.5	0.9	8.5 + 40.0	4.9
Olive oil	28.4	2.8	28.4	2.8
Safflower oil	2.7	0.3	2.7	0.3
Total	132.2	13.2	172.2	17.2
**Water**	**867.8**	86.8	**827.8**	82.8
Total	1000.0		1000.0	

^a^ Originally choline bitartrate, replaced by choline chloride

^b^ Originally Xanthan gum, replaced by Guar gum.

**FIGURE 3:**
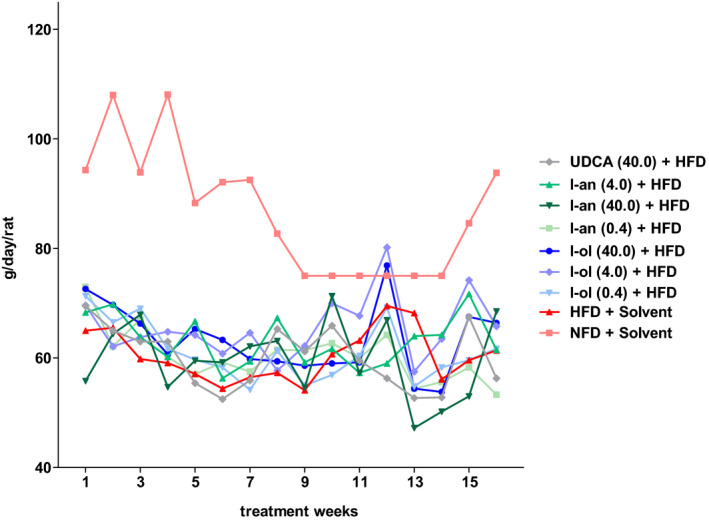
Food consumption dynamics: Rats from the disease group (n = 8) and treatment groups (n = 6 to 8 were offered HFD permanently (ad libitum). Each point represents the arithmetic mean value. For a better view, the error bars have been omitted

Both LDC diets were in a liquid form as produced by ssniff‐Spezialdiäten GmbH, Soest (Germany). The feed was provided over the entire experimental period of 16 weeks; however, the corresponding experimental substances were only administered in the last four weeks (Figure 3).

The experimental substances I‐ol, I‐an, and UDCA were dissolved in a 0.8% aqueous HPMC gel to obtain the following concentrations: (1) I‐ol at 0.4, 4, and 40 mg/kg body weight (b.w.), (2) I‐an at 0.4, 4, and 40 mg/kg b.w., and (3) UDCA at 40 mg/kg b.w. The Group‐2 and −3 rats received only HPMC as placebo. Each test encompassed 6–8 rats that were put up in a separate cage. The rats were fed ad libitum, and their intake per cage was controlled daily. In order to ensure an exact daily reproducible dosage, the experimental substances or only HPMC was administered intragastrically via oral gavage. Basically, the drug therapy of a chronic disease is the more efficient the higher the adherence of the affected patients. This is generally higher with peroral administration than with any other form of application. At the end of the experiment, the animals were sacrificed under fasting conditions, by aorta dissection and exsanguination, after anesthetizing with an intraperitoneal injection of 5% pentobarbital solution.

### 2.3 Acute toxicity study

The study was performed in compliance with (a) the “Good Laboratory Practice” Regulations of the EC, Germany, in the “Chemikaliengesetz” (Chemicals Act), current edition and (b) the United States Food and Drug Administration Good Laboratory Practice Regulations.

The following regulations were considered: (1) “OECD Principles of Good Laboratory Practice” Document Nos. 1 and 13 of ENV/MC/CHEM (98) 17 and ENV/JM/MONO (2002) 9, respectively; and (2) Japanese Guidelines for Nonclinical Studies of Drugs Manual 1995; Guidelines for Toxicity Studies of Drugs, Japanese Ministry of Health and Welfare.

In total, 90 male CD^®^/Crl:CD(SD) rats from Charles River Laboratories, Deutschland GmbH, were used after randomization. Five rats were selected for each concentration of the corresponding experimental substance. This resulted in 30 animals per experimental substance and a total of 18 experimental groups. In addition, a control group was formed with five rats receiving only the HPMC gel. At the time of application of the test substances, the animals had a body weight of 203–246 g and age in the range of 49–51 days.

The standard ssniff^®^ R/M‐H V 1534 diet (ssniff‐Spezialdiäten GmbH, 59494 Soest) was used as feed. Feeding was stopped approximately 16 h before the start of test substance application, whereas tap water continued to be available ad libitum. Granulated structural wood was used as bedding material in the cages; the material was changed and the cages were cleaned twice a week. During the 14‐day observation period, the animals were housed in groups of 2–3 in Makrolon^®^ cages (type Ill) at a room temperature of 22 ± 3°C (maximum range) and relative humidity of 55 ± 15% (maximum range). The rooms were illuminated (approximately 150 lux from a ceiling height of approximately 1.50 m) and darkened for 12 h in turns.

Each animal was kept under observation, so that any behavior deviating from the norm could be systematically recorded. Observations were made before the peroral application of the respective active substance, immediately after, and 5, 15, 30, and 60 min and 3, 6, and 24 h after the start of application. This was followed by observation at least once a day over a period of 14 days. We focused on the changes in the skin and fur, the eyes and mucous membranes, respiration, circulation, the autonomous and central nervous systems, somatomotor activity, and behavior patterns. They were also observed for possible tremors, convulsions, salivation, diarrhea, lethargy, sleep, and coma. Mortality checks were performed at least once a day to minimize the loss of animals during the study. The time of death was recorded as accurately as possible. Individual body weights were recorded before the administration of experimental substances and, subsequently, at weekly intervals, until the end of the study and at death. Weight changes were calculated when survival exceeded 1 day.

At the end of the experiments, all surviving animals were sacrificed, dissected, and examined macroscopically. All notable pathological changes were recorded. A microscopic examination of all organs showing obvious lesions was performed on animals that survived for 24 h or longer. Autopsy and macroscopic inspection of prematurely deceased animals were performed as soon as possible after death.

### 2.4 Liver histopathology

Pieces of liver tissue measuring 1 × 2 × 0.5 mm were chosen randomly and fixed for a week in the mixture of 1% formaldehyde and 1% paraformaldehyde in Sorensen's phosphate buffer (pH 7.4) (Morphisto GmbH, Frankfurt/Main). After this the pieces were kept in 2.5% glutaraldehyde in same buffer, rinsed and then post‐fixed in 1% solution of osmium tetroxide on the Sorensen's phosphate buffer (pH 7.4). After fixation, the liver samples were dehydrated and embedded in the pre‐polymerized mixture of methyl methacrylate and butyl methacrylate (1:4, v/v) containing 2,4‐dichlorbenzoyl peroxide as a catalyst.

Sections of 0.5–1.0 µm thickness were stained using Asur II, methylene blue, and alkaline fuchsin. A semi‐quantitative evaluation of the sections was performed according to the procedure described by Koppe et al. (Koppe et al., 2004).

### 2.5 Synthesis of ω‐imidazolyl‐alkyl derivatives

12‐Imidazolyl‐1‐dodecanol (I‐ol) and 1‐Imidazolyldodecane (I‐an) were prepared based on a protocol published by Lu et al. (Lu et al., 1997). 12‐bromo‐1‐dodecanol and imidazole were mixed at a molar ratio of 1:3 and stirred for 5 h at 80°C. The resulting product mixture was subsequently extracted with the solvent dichloromethane/water. The organic phase was dehydrated using Na_2_SO_4_ and evaporated thereafter. I‐ol was recrystallized from benzene/n‐hexane.

I‐an was synthesized from 1‐bromododecane and imidazole according to the aforementioned procedure. As a deviation, it was recrystallized from n‐hexane.

Phosphatidylcholine was converted to *O*‐phosphorylisourea under acidic conditions in the presence of dicyclohexylcarbodiimide. I‐ol was added to the reaction mixture, which caused dicyclohexylurea to settle. For this reaction to succeed, 4‐diethylaminopyridine was used as the catalyst. The reaction mechanism was similar to that of Steglich esterification, where dicyclohexylcarbodiimide is used to esterify an organic acid with an alcohol.

### 2.6 Oxidative stress in the liver

The formation of ROS in the rat liver was measured using NADPH‐induced chemiluminescence as described by Müller‐Peddinghaus et al. (Muller‐Peddinghaus, 1987). Luminol and lucigenin were used for detecting the superoxide radical anion (SRA) and hydrogen peroxide. Liver pieces were homogenized in 0.1 M sodium phosphate buffer, pH 7.4; the homogenates were centrifuged at 9,000 × *g* at 0°C; and aliquots were used for subsequent measurements. Reduced glutathione (GSH) was estimated via a modified Ellman's method (Ellman, 1959), in which 0.2 mL of the liver homogenate supernatant was added to 1.6 mL of an EDTA/sodium phosphoric acid solution and the absorbance noted at 412 nm against blank values. GSH solution produced immediately before application served as the standard. The end products of the lipid peroxidation reaction were equivalent to the thiobarbituric acid‐reacting substances (TBARS), and their concentration was measured according to the method described by Buege et al. (Buege & Aust, 1978). Enzymatic activities of microsomal glutathione reductase (GR) and cytosolic glutathione peroxidase (GPx) were measured according to the methodology of Buko et al. (Buko et al., 2002). Catalase activity was determined according to the spectroscopic methodology described by Beers et al. (Beers & Sizer, 1952) using hydrogen peroxide as the substrate.

### 2.7 NASH biomarker levels in serum samples

The activities of the liver enzymes aspartate aminotransferase (AST), alanine aminotransferase (ALT), alkaline phosphatase (AP), and γ‐glutamyl transferase (γ‐GT) as well as bilirubin and triglyceride levels in the serum were determined as described by the instructions of BIO‐LA‐tests kit (Erba Lachema, Brno, Czech Republic).

The concentrations of adiponectin and TNFα were measured using ELISA kits sold by BioCat GmbH (Heidelberg, Germany), whereas the one for determining leptin level was procured from BioVendor Laboratory Medicine Inc. (Brno, Czech Republic). Insulin concentration was determined using the radioimmunoassay kit RIO‐insulin‐PG‐J125 (IBOCH, Minsk, Belarus). Serum glucose level was determined based on the “Accu‐Check Active blood glucose” monitoring method developed by Roche‐Diagnostics.

### 2.8 End products of fat metabolism

Extraction of lipids was performed using a solvent composed of chloroform and methanol (2:1 v/v). Thin‐layer chromatography (TLC) was used to separate off neutral lipids and phospholipids. Silicagel G 60 plates (0.25 mm thick) were used. The lipids were separated by ascending chromatography in a system composed of hexane:diethylic ether:glacial acetic acid (73:25:2; v/v/v). They were visualized by iodine vapor and the spots corresponding to triglycerides and cholesterol were scraped off and eluted with methanol for spectrophotometric evaluation. Triglyceride and cholesterol levels were determined using the BIO‐LA‐test kit (Erba Lachema, Brno, Czech Republic). Phospholipid concentration was determined by measuring the levels of phosphomolybdate using an inorganic phosphate reagent containing molybdate (Findlay & Evans, 1987). VLDL level was determined by using the manganese precipitation method (Burstein & Scholnick, 1973) after partial modification (Hirano et al., 2003).

### 2.9 Fibrosis‐related parameters of the liver

Liver tissue sections were subject to the Mallory‐Azan (Heidenhain Azan) staining method for visualizing the connective tissues. This method was a modification of the original Mallory staining protocol, wherein azocarmin with a mixture of aniline blue and orange G was used. In this method, the collagen tissue and basophilic granules turn blue, the muscle tissue and acidophilic granules turn orange to red, and the cell nuclei and cytoplasm turn red. Elastic fibers remain unstained; however, they might turn yellow or pink. The proportion of positively stained liver tissue to the total area of the histological slide was determined quantitatively.

### 2.10 Statistical analysis

We used SPSS (IBM SPSS Statistics, Version 22, USA) for performing statistical analysis and GraphPad PRISM (Version 5, GraphPad Software, Inc., USA) for generating the diagrams.

**FIGURE 4:**
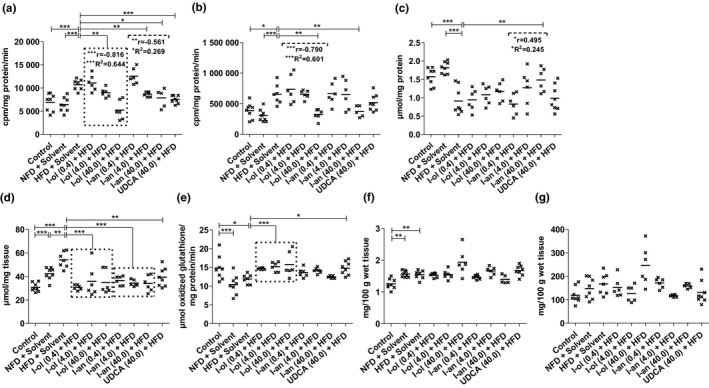
. In‐vivo ROS status in the rat liver: Rats from the disease group (n = 8) and treatment groups (n = 6 to 8) were offered HFD permanently (ad libitum). (a) SRA level (b) Hydrogen peroxide level (c) GSH level (d) TBARS level (e) GR activity. (f) GPx activity. (g) Catalase activity Bonferroni adjustment for multiple comparisons reduced the significance level to *p < 0.0167, **p < 0.00333, and ***p < 0.000333. Dose‐dependent effects were specified by Pearson's correlation coefficient ‘r’, and linear regression analysis was represented by the coefficient of determination ‘R^2^’ with + *p* < 0.05, ^++^ *p* < 0.01, and ^+++^ *p* < 0.001

**FIGURE 5:**
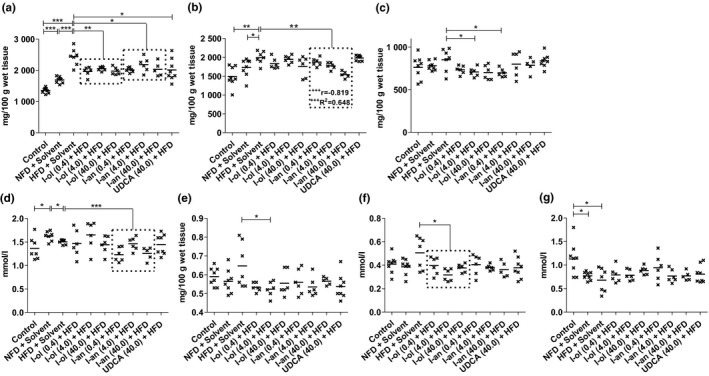
Liver and serum fat content: Rats from the disease group (n = 8) and treatment groups (n = 6 to 8) were offered HFD permanently (ad libitum). (a) Level of hepatic triglycerides (b) Level of hepatic phospholipids (c) Hepatic cholesterol level. (d) Level of serum triglycerides (e) Serum VLDL level. (f) Serum LDL cholesterol level (g) Serum HDL cholesterol level. Bonferroni adjustment for multiple comparisons reduced the significance level to * *p* < 0.0167, ** *p* < 0.00333, and *** *p* < 0.000333. Dose‐dependent effects were specified by Pearson's correlation coefficient ‘r’ and linear regression analysis by the coefficient of determination ‘R^2^’ with + *p* < 0.05, ^++^ *p* < 0.01, and ^+++^ *p* < 0.001

**FIGURE 6:**
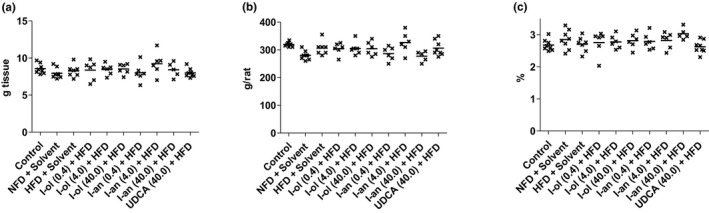
Body and Liver weight: Rats from the disease group (n = 8) and treatment groups (n = 6 to 8) were offered HFD permanently (ad libitum). The presented results represent an end‐point measurement. Each point represents an individual value of a pathologically relevant parameter in a single rat. The mean value is displayed as a horizontal line. (a) Liver weight (b) Final body weight (c) Liver‐to‐body weight ratio

**FIGURE 7:**
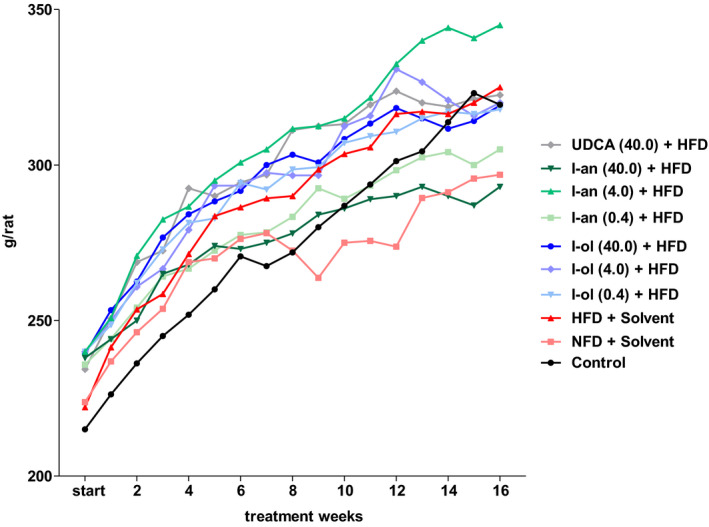
Body weight dynamics: Rats from the disease group (n = 8) and treatment groups (n = 6 to 8) were offered HFD permanently (ad libitum). Each point represents the arithmetic mean value. For a better view, the error bars have been omitted

**FIGURE 8:**
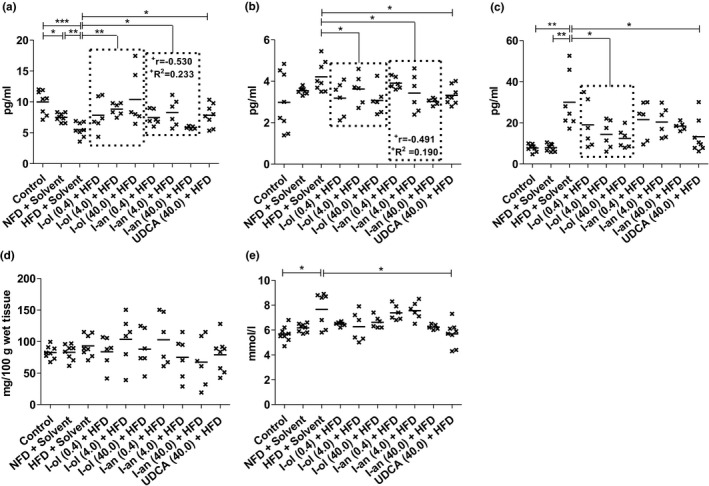
Adipocytokines and glucose metabolism: Rats from the disease group (n = 8) and treatment groups (n = 6 to 8) were offered HFD permanently (ad libitum). (a) Serum adiponectin level (b) Serum leptin level (c) Serum TNF‐α level (d) Serum insulin concentration (e) Serum glucose level. Bonferroni adjustment for multiple comparisons reduced the significance level to * *p* < 0.0167, ** *p* < 0.00333, and *** *p* < 0.000333. Dose‐dependent effects were specified by Pearson's correlation coefficient ‘r’ and linear regression analysis by the coefficient of determination ‘R^2^’ with ^+^ *p* < 0.05, ^++^ *p* < 0.01, and ^+++^ *p* < 0.001

**FIGURE 9:**
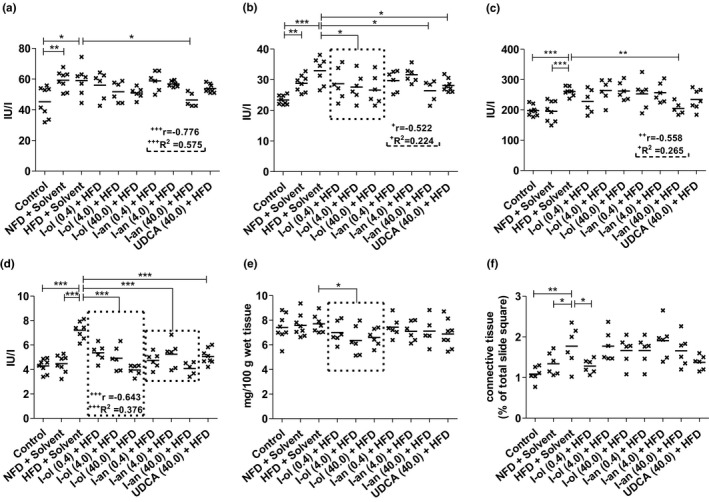
Liver enzyme activity, cholestase, and fibrosis‐related parameters: Rats from the disease group (n = 8) and treatment groups (n = 6 to 8) were offered HFD permanently (ad libitum). (a) ALT enzymatic activity (b) AST enzymatic activity (c) AP enzymatic activity (d) γ‐GT enzymatic activity (e) Serum bilirubin level (f) Area of connective tissue in the liver. Bonferroni adjustment for multiple comparisons reduced the significance level to * *p* < 0.0167, ** *p* < 0.00333, and *** *p* < 0.000333. Dose‐dependent effects were specified by Pearson's correlation coefficient ‘r’, and linear regression analysis by the coefficient of determination ‘R^2^’ with ^+^ *p* < 0.05, ^++^ *p* < 0.01, and ^+++^ *p* < 0.001

**FIGURE 10:**
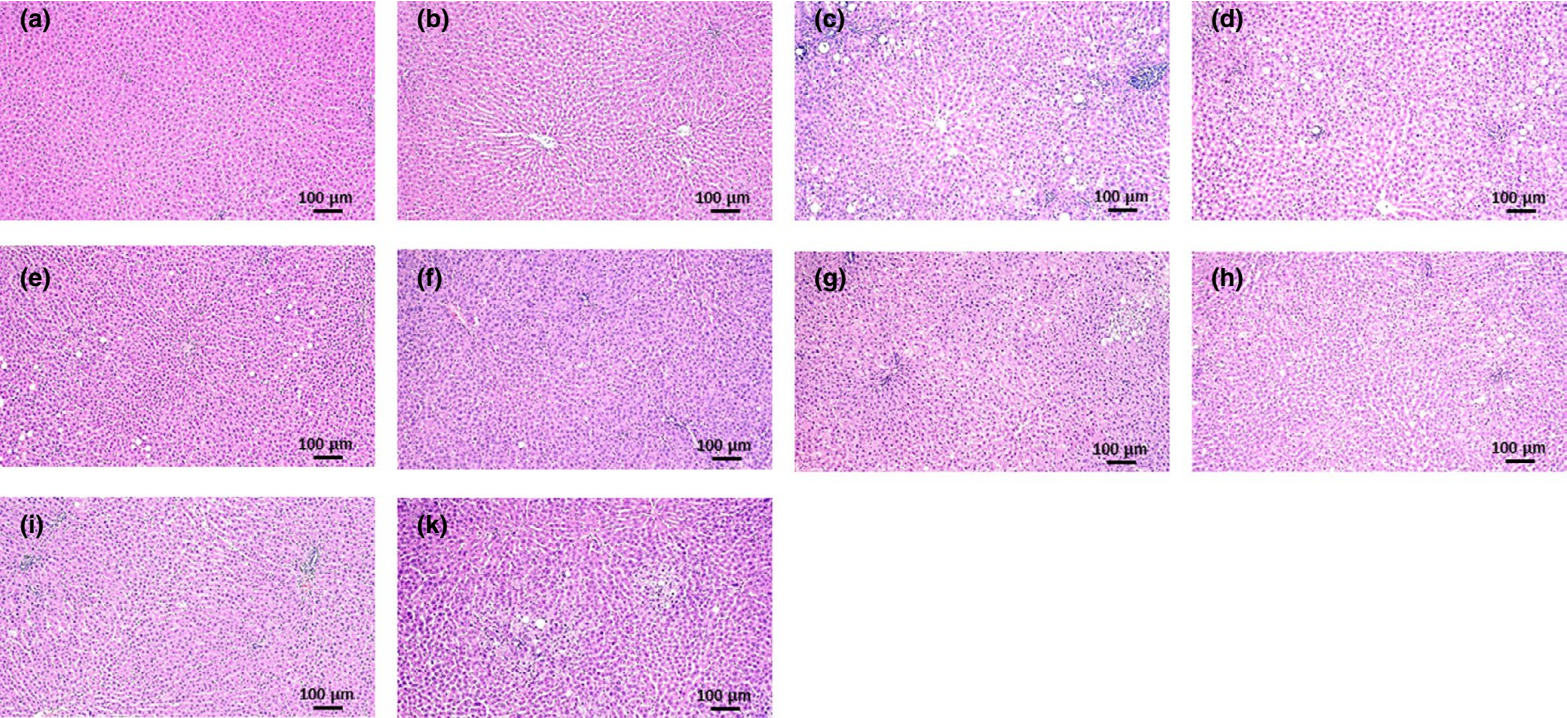
Representative histological images of liver sections: (a) Control, (b) NFD +solvent, (c) HFD +solvent, (d) I‐ol (0.4 mg/kg b.w.) + HFD, (e) I‐ol (4 mg/kg b.w.) + HFD, (f) I‐ol (40 mg/kg b.w.) + HFD, and (g) UDCA (40 mg/kg b.w.) + HFD. To avoid redundancy, illustrations of the I‐an groups were omitted. Nine sections of each liver were prepared, with six or eight animals per group. The sections are stained with H.E. (original magnification: 100×. Each scale bar indicates 100 μm)

The outcome of the experimental animal study is presented as individual points of the dependent variables (i.e. the calculated pathological values) plotted according to the average values shown as a horizontal line (Figures 1, 2, 3, 4, 5, 6, 7, 8, 9, 10). The statistics were calculated based on one‐way ANOVA followed by complex custom/planned contrasts, the results of which were structured as follows: statistical differences across the (a) diet groups (groups 1–3), (b) I‐ol groups (group 4–6) and disease group (group 3), (c) I‐an groups (groups 7–9) and disease group (group 3), (d) UDCA (group 10) and disease group (group 3). A simple contrast analysis was performed only if no statistically significant difference was observed between the therapeutic groups (I‐ol or I‐an) as a whole and the disease group. Thus, we have been able to determine the statistical relevance of each inhibitor concentration individually.

Owing to a clear assumption regarding the effect of the different animal diets (groups 1–3) and tested compounds (groups 4–10), the hypotheses were regarded as unilateral. In all cases, a highly conservative correction of a type I error in multiple comparisons was performed by the Bonferroni adjustment, which lowered the significance level to *p < 0.0167 (significant), **p < 0.00333 (highly significant), and ***p < 0.000333 (very highly significant).

Pearson's correlation and linear regression were applied to rate‐ and dose‐dependent effects of I‐ol and I‐an, revealing the following statistical parameters: ^+^ *p* < 0.05 (significant), ^++^ *p* < 0.01 (highly significant), and ^+++^ *p* < 0.001 (very highly significant); 0.10 ≤ ǀrǀ < 0.30 (moderate correlation), 0.30 ≤ ǀrǀ < 0.50 (moderate correlation), and ǀrǀ ≥ 0.50 (strong correlation); and 0.0196 ≤ R^2^ < 0.1300 (small effective power), 0.1300 ≤ R^2^ < 0.2600 (moderate effective power), and R^2^ ≥ 0.2600 (strong effective power).

## 3 RESULTS

The following section does not explicitly address the respective significance level with regard to the therapeutic effect. This can be seen in the corresponding figures (Figure 4‐9). Only the reference to an existing dose–response relationship and the absence of a statistically significant difference was noted.

### 3.1 ROS status

SRA level (Figure 4a) was increased in HFD‐fed rats by 55% and 67%, respectively, compared to that in the control groups. I‐ol and I‐an administration caused an average decrease in SRA level by 21% and 23%, respectively, compared to that in the disease group. UDCA induced a 30% decrease of SRA. Thus, the effect of HFD on the SRA level was reduced on average by 60% and 65% through the application of I‐ol and I‐an, and by 84% through the application of UDCA respectively. The highest concentration of I‐ol reduced the effect of HFD on the SRA level to 24% below the reference value of control group 1. I‐an at medium and highest concentrations reduced the SRA level by 54% and 75%, respectively. I‐ol showed a strong, very highly significant (*p* < 0.001), linear, dose‐dependent effect, whereas I‐an revealed a weak, significant (*p* < 0.05), linear, dose‐dependent effect. Hydrogen peroxide level (Figure 4b) was increased in HFD‐fed rats by 69% and 115% compared to that in the control groups 1 and 2 respectively. The highest concentrations of I‐ol and I‐an caused a decrease of 50% and 43%, respectively, in hydrogen peroxide level compared to that in the disease group. This not only eliminated the effect of HFD on hydrogen peroxide level but also decreased it by 16% and 4%, respectively, below the reference values measured in group 1. I‐ol showed a strong, very highly significant (*p* < 0.001), linear, dose‐dependent effect; UDCA displayed no significant effect. Similarly, GSH level (Figure 4c) was reduced by 42% and 50% respectively in the HFD‐fed rats. I‐an at its highest concentration increased the GSH level by 63% and thus reduced the effect of HFD by 87% compared to that in the disease group. Overall, the I‐an therapy groups showed a weak, significant (*p* < 0.05), linear, dose‐dependent effect. I‐ol exhibited a non‐significant trend toward an increasing GSH level; UDCA was overall ineffective.

The TBARS level (Figure 4d) was 76% and 28% higher in HFD‐fed rats than in control groups 1 and 2 respectively. Control group 2 had a 38% higher level than control group 1. I‐ol and I‐an induced an average decrease of 38% and 35%, respectively, and UDCA was responsible for a 27% decrease compared to that in the disease group. Thus, the effect of HFD on the TBARS level was reduced on an average by 87% and 82% after I‐ol and I‐an administration, and by 63% after UDCA administration respectively. GR activity (Figure 4e) was 20% lower in the HFD‐fed rats than in control group 1, whereas it was 14% higher in the HFD‐fed rats than in control group 2, which showed a decrease of 30% compared to that in control group 1. Compared to the disease group, I‐ol increased the GR activity by 28%. UDCA increased it by 24%. Thus, I‐ol and UDCA nullified the effect of HFD and increased the GR activity by 113% and 99%, respectively, on an average, whereas I‐an showed no effect.

GPx activity (Figure 4f) in HFD‐fed rats was 22% higher than that in control group 1. However, group 1 showed no difference compared to control group 2, which exhibited 24% higher activity than control group 1. None of the experimental compounds showed a statistically relevant effect on catalase activity in all the groups (Figure 4g).

### 3.2 Lipid balance

Liver triglyceride levels (Figure 5A) were increased in HFD‐fed rats by 80% and 44%, respectively, compared to those in control groups 1 and 2. Control group 2 exhibited a concentration 25% higher than that in control group 1. I‐ol and I‐an induced an average decrease of 18% and 15%, respectively, in liver triglyceride levels compared to that in the disease group. UDCA induced a decrease of 17%. Thus I‐ol and I‐an reduced the effect of HFD by an average of 41% and 33%, respectively, and UDCA by 39%. Liver phospholipid levels (Figure 5b) were increased by 33% and 15% in the HFD‐fed rats compared to those in control groups 1 and 2 respectively. I‐an caused an average decrease of 13% in these levels compared to those in the disease group. This corresponded to a 53% reduction of the effect of HFD, with the highest concentration of I‐an reducing it only to 3%, which is above the reference value measured in group 1. While a strong and very highly significant (*p* < 0.001), linear, dose‐dependent effect was observed here, I‐ol and UDCA showed no therapeutic effect.

Hepatic cholesterol (Figure 5c) level showed no difference between the two control groups and the disease group. Although all the three concentrations of I‐ol caused a comparable reduction in hepatic cholesterol level, only the medium concentration of I‐ol and the lowest concentration of I‐an could completely reduce the effect of HFD. The medium dose of I‐ol and low dose of I‐an reduced the liver cholesterol level by 17% and 18%, respectively, compared to that in the disease group. This corresponded to a decrease in cholesterol levels by 7% and 8%, respectively, which is below the reference value measured in group 1. Serum triglyceride levels (Figure 5d) in control group 2 were increased by 19% compared to those in control group 1, whereas the disease group exhibited a 7% lower level than control group 2. I‐an showed an average decrease of 12% compared to that in the disease group. Thus, I‐an not only completely reversed the effect of HFD but also reduced it by an average of 4%, which is below the reference value measured in group 1; however, I‐ol and UDCA showed no therapeutic effect. Serum VLDL level (Figure 5e) showed no difference between the two control groups and the disease group. Although all three experimental compounds exhibited a trend toward decreasing the serum VLDL level, only the medium concentration of I‐ol reduced it by 19%. This corresponded to a complete reduction of the effect of HFD far below the reference value measured in group 1. The level of serum LDL cholesterol (Figure 5F) showed no difference between the two control groups and the disease group. I‐ol induced an average decrease of 28% and thus not only completely reversed the effect of HFD but also lowered it by an average of 11% below the reference value measured in group 1. Both I‐an and UDCA showed a non‐significant trend toward lowering the serum LDL level. Serum HDL cholesterol in the disease group was reduced by 40% compared to that in control group 1, which exhibited a 32% higher concentration than control group 2. None of the experimental compounds showed a statistically relevant effect on the concentration of serum HDL cholesterol (Figure 5g).

In summary, I‐ol and I‐an exhibited a therapeutic effect with regard to nearly all measured parameters, whereas UDCA only had a corresponding effect on the hepatic triglyceride levels. I‐an reduced hepatic phospholipid levels in a concentration‐dependent manner. The NFD group showed an increase in the liver and serum triglyceride levels and a decrease in the HDL cholesterol levels. None of the three compounds exhibited a therapeutic effect on the HDL cholesterol levels. There was no statistically significant difference between the disease and control groups with regard to the levels of liver cholesterol and serum VLDL and LDL cholesterol. This finding was supported by the lack of increase in the body and liver weights in the disease group (Figures 5, 6).

### 3.3 Adipocytokines (adiponectin, leptin, and TNF‐α) and glucose metabolism

Serum adiponectin (Figure 8a) was reduced by 45% and 27%, respectively, in the HFD‐fed rats compared to that in control groups 1 and 2; control group 2 exhibited a level 25% lower than control group 1. I‐ol and I‐an induced an average increase of 64% and 31%, respectively, and UDCA of 42% compared to that the disease group. Thus, I‐ol and I‐an reduced the effect of HFD by an average of 78% and 38%, respectively, with I‐ol at its highest concentration increasing it to 4% above the reference value measured in control group 1; UDCA elevated the concentration by 52%. I‐an showed a weak, significant (*p* < 0.05) linear dose‐dependent effect. Serum leptin (Figure 8b) was non‐significantly elevated by 41% and 19% compared to that in control groups 1 and 2, respectively. I‐ol and I‐an induced an average decrease of 22% and 18%, respectively compared to that in disease group. UDCA showed a decline of 21%. Thus, both I‐ol and I‐an decreased the effect of HFD by 75% and 62%, respectively, on an average, with their highest concentrations reducing the effect of HFD to 3% and 1%, respectively, which is above the reference value measured in control group 1. UDCA reduced leptin concentration by 72%, whereas I‐an showed a weak yet significant (*p* < 0.05) linear dose‐dependent effect. Serum TNF‐α level (Figure 8c) was increased by 286% and 280%, respectively, in the HFD‐fed rats compared to that in control groups 1 and 2. I‐ol induced an average decrease of 49% in serum TNF‐α level compared to that in the disease group. On the other hand, UDCA induced a decrease of 56%. Thus, both I‐ol and UDCA reduced the effect of HFD by an average of 66% and 75% respectively. The effect of I‐an on decreasing TNF‐α level was not significant. Serum insulin level (Figure 8d) showed no difference across all groups. Serum glucose level (Figure 8e) in the HFD‐fed rats was increased by 35% compared to that in control group 1. UDCA decreased the level by 26%, which lead to a 100% reversal of the effect of HFD.

### 3.4 Liver enzymes, cholestase, and fibrosis‐related parameters

In this study, the activities of ALT and AST increased by 41% and 30%, respectively, compared to those in control group 1, whereas the De‐Ritis quotient was 1.8, indicating severe liver damage in the disease group.

ALT activity (Figure 9a) in the HFD‐fed rats was increased by 41% compared to that in control group 1, which exhibited 23% lower enzymatic activity than in control group 2. I‐ol and I‐an, at their highest concentrations, caused a 16% and 20% average decrease in enzymatic activity compared to that in the disease group, corresponding to a 55% and 66% average reduction of the effect of HFD respectively. I‐an showed a weak, significant (*p* < 0.05), linear, dose‐dependent effect. UDCA lowered the enzymatic activity by 15%, corresponding to a 50% reduction in the effect of HFD. AST activity (Figure 9b) was increased by 30% in the HFD‐fed rats than that in control group 1, which exhibited 31% less enzymatic activity compared to that in control group 2. The highest concentration of I‐an caused a 21% decrease in enzymatic activity and prevented the effect of HFD almost completely by a 92% reduction compared to that in the disease group. It also showed a moderate, very highly significant (*p* < 0.001), linear, dose‐dependent effect; I‐ol and UDCA showed no therapeutic effect. AP activity (Figure 9c) in the HFD‐fed rats was increased by 32% and 33% compared to that in control groups 1 and 2 respectively. The highest concentration of I‐an showed a 21% average decrease in enzymatic activity, compared to that in the disease group, thus reducing the effect of HFD by 89%. However, it caused a weak but significant (*p* < 0.05), linear, dose‐dependent effect; I‐ol and UDCA showed no therapeutic effect. γ‐GT activity (Figure 9d) was increased by 68% and 61% in HFD‐fed rats compared to that in control groups 1 and 2 respectively. I‐ol and I‐an showed an average decrease in enzymatic activity of 34% and 35%, respectively, and UDCA of 30% compared to that in the disease group. Thus, I‐ol and I‐an reduced the effect of HFD by 84% and 86%, respectively, and UDCA by 73%. I‐ol showed a moderate but very highly significant (*p* < 0.001), linear, dose‐dependent effect.

Serum bilirubin level (Figure 9e) showed no difference between the two control groups and the disease group. I‐ol showed an average decrease of 14% in serum bilirubin level compared to that in the disease group, corresponding to an average of more than 10% below the levels measured in the disease group and the two control groups. I‐an and UDCA showed no therapeutic effect. The area of connective tissue in the liver (Figure 9f) was increased by 63% and 32%, respectively, in the HFD‐fed rats compared to that in control groups 1 and 2. I‐ol showed a decrease of 28% at its lowest concentration compared to the disease group, thus reducing the effect of HFD by 72%. I‐an and UDCA showed no therapeutic effect.

In summary, the significant increase in the activities of all the four liver enzymes because of HFD was almost completely nullified by the tested compounds. The maximum effect occurred at the lowest concentrations of I‐ol and I‐an, which showed a weak or moderate, linear, dose‐dependent effect.

### 3.5 Histopathology

**FIGURE 11:**
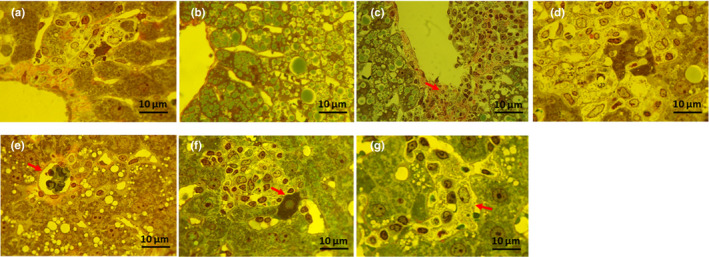
Additional histopathological images: The magnification of the objective lens was 100‐fold and that of the ocular lens was 10‐fold, thus making the total magnification 1000‐fold. (a) Healthy animals from the NFD group; (b)–(d) animals from the HFD‐treated group; (b) hepatocytes overloaded with manifold lipid droplets; (c) pronounced macrovesicular steatosis and spacious necrotic area near the central vein. The nuclei of the macrophages and lymphocytes are visible (arrows); (d) necrotic focus. Hepatocytes lysed by macrophages and lymphocytes are present in the center. (e) Rat treated with UDCA (40 mg/kg b.w.), inflammatory area around a hepatocyte destroyed by a large lipid droplet (arrow); (f) rat treated with I‐ol (40 mg/kg b.w.), apoptotic cell (arrow) on the border of a necrotic focus in the portal area; (g) rat treated with I‐an (40 mg/kg b.w.), hypertrophic macrophage (arrow) and clustering of lymphocytes in a liver sinusoid

The HFD‐fed rats developed macrovesicular steatosis (Figure 10c and Figure 11b,c) with focal necrosis (Figure 10c, Figure 11c) and intralobular lymphocytic infiltration. Both ROS inhibitors and UDCA improved liver histology in the animals with NASH, decreasing steatosis, ballooning, and necrosis (Figure 10d‐k, Table 3).

**TABLE 3:** Semi‐quantitative evaluation of the H.E.‐stained liver slices: Semi‐quantitative eval‐uation of the liver slices, shown representatively in Figure 5, was performed according to the method described by Koppe et al., 2004. [20]

	Control	NFD +Solvent	HFD +Solvent	UDCA (40.0) + HFD	I‐ol (0.4) + HFD	I‐ol (4.0) + HFD	I‐ol (40.0) + HFD	I‐an (0.4) + HFD	I‐an (4.0) + HFD	I‐an (40.0) + HFD
Steatosis^a^	0	0	1	0–1	0–1	0	0–1	0–1	0–1	0–1
Balooning^a^	0	0	1	0	1	1	1	0–1	1	0–1
Inflammation^b^	0	0	2	1	1–2	1	1	1	1	1

Steatosis and ballooning: 0, none; 1, ≤25%; 2, 26–50%; 3, ≥51–75% of liver parenchyma.

^b^ Inflammation: 0, none; 1, <5 signs of inflammation; 2, >5 signs of inflammation in the microscopic field at 40x magnification.

### 3.6 Acute toxicity study

I‐ol (Table 4) showed no acute toxicity up to a concentration of 1000 mg/kg b.w. At this maximum dose, reduced motility was observed, with reduced muscle tone, ataxia, and dyspnea, whereas no inhibition of body weight gain or necropsy was detected further on. None of the 30 rats died; therefore, the lowest lethal dose and the LD_50_ dose (14 days) had to be greater than 1000 mg/kg b.w.

**TABLE 4:** Results of the acute toxicity study of I‐ol

Symptoms	Control	I‐an [mg/kg b.w., p.o.]					
		10	50	100	250	500	1000
	Males	Males	Males	Males	Males	Males	Males
	(n = 5)	(n = 5)	(n = 5)	(n = 5)	(n = 5)	(n = 5)	(n = 5)
Reduced motility	None	None	None	None	None	None	None
Ataxia	None	None	None	None	None	None	None
Reduced muscle tone	None	None	None	None	None	None	None
Dyspnoea	None	None	None	None	None	None	None
Ptosis	None	None	None	None	None	None	None
Chromodacryorrhea	None	None	None	None	None	None	None
Piloerection	None	None	None	None	None	None	None
Mortality							
Within 6 h	0	0	0	0	0	0	0
Within 24 h	0	0	0	0	0	0	0
Within 7 h	0	0	0	0	0	2	1
Within 14 h	0	0	0	0	0	2	1
Mean body weight [g]							
Start	215.8	235.6	234.2	232.4	223.0	237.0	235.0
After 7 d	269.8	293.2	296.4	283.6	266.2	288.7	270.3
End	312.0	328.4	329.2	311.8	296.0	328.0	320.0
Mean body weight gain [%]							
After 7 d	+25.0	+24.4	+26.6	+22.0	+19.4	+21.8	+15.0
End	+44.6	+39.4	+40.6	+34.2	+32.7	+38.4	+36.2
Inhibition of body weight gain	None	None	None	None	None	None	None
Necropsy findings	None	None	None	None	None	None	None

+ = Slight/observed

++ = Moderate

In brackets: Number of animals affected

The application of I‐an (Table 5) led to the premature death of three rats; two died at a dose of 500 mg/kg b.w. after 6 days and one died at 1000 mg/kg b.w. after 5 days. Thus, the lowest lethal dose was 500 mg/kg b.w., whereas the LD_50_ dose (14 days) had to be greater than 1000 mg/kg b.w.

**TABLE 5:** Results of the acute toxicity study of I‐an

Symptoms	Control	I‐ol (mg/kg bw, p.o.)					
		10	50	100	250	500	1000
	Males	Males	Males	Males	Males	Males	Males
	(n = 5)	(n = 5)	(n = 5)	(n = 5)	(n = 5)	(n = 5)	(n = 5) +
Reduced motility	None	None	None	None	None	None	3–6 h (5) +
Ataxia	None	None	None	None	None	None	3–6 h (5) +
Reduced muscle tone	None	None	None	None	None	None	3‐6 h (5) +
Dyspnoea	None	None	None	None	None	None	3–6 h (5)
Ptosis	None	None	None	None	None	None	None
Chromodacryorrhea	None	None	None	None	None	None +	None
Piloerection	None	None	None	None	None	2d (5)	None
Mortality							
Within 6 h	0	0	0	0	0	0	0
Within 24 h	0	0	0	0	0	0	0
Within 7 d	0	0	0	0	0	0	0
Within 14 d	0	0	0	0	0	0	0
Mean body weight (g)							
Start	215.8	221.6	227.8	230.0	223.0	228.0	221.6
After 7 d	269.8	259.2	278.4	272.6	266.8	294.2	271.2
End	312.0	288.0	310.4	305.8	299.2	334.2	302.6
Mean body weight gain (%)							
After 7 d	+25.0	+17.0	+22.2	+18.5	+19.6	+29.0	+22.4
End	+44.6	+30.0	+36.3	+33.0	+34.2	+46.6	+36.6
Inhibition of body weight gain	None	None	None	None	None	None	None
Necropsy findings	None	None	None	None	None	None	None

+ = slight/observed

++ = moderate

In brackets: Number of animals affected

A summary of the study results is depicted in Table 6.

**TABLE 6:** Summary of the acute toxicity study with ω‐imidazolyl‐alkyl derivatives

Toxicological parameters	I‐ol	I‐an
No Observed Adverse Effect Level	500	250
(mg/kg bw)		
No‐Observed‐Effect Level	1000	500
(mg/kg bw)		
Lowest lethal dose	>1000	500
(mg/kg bw)		
LD_50_(14 d)	>1000	>1000
(mg/kg bw)		
Toxic signs		
250 mg/kg bw	None	None
500 mg/kg bw	None	Mortality
1000 mg/kg bw	Reduced motility and muscle tone, ataxia, dyspnoea	Mortality
Macroscopic necropsy findings	None	None

+ = slight/observed

++ = moderate

In brackets: Number of animals affected

## 4 DISCUSSION

We previously showed, in an animal model, that I‐ol and I‐an, two new CYP2E1 enzyme inhibitors, are excellent drug candidates for the treatment of ASH (S7 Fig) (Diesinger et al., 2020). In the present study, we investigated whether they are as suitable for NASH therapy.

The enzymatic activities of ALT and AST are increased in the serum of patients with steatosis and NASH. However, this observation is not mandatory for diagnosis (Vernon et al., 2011). The diagnostic value of ALT for NASH is controversial and based on unclear study results (Gauthier, 2006). In the present study, the activity of ALT in the HFD‐fed rats was found to be higher than that in the NFD‐fed rats. This observation was supported by the results of another study, in which the activity of ALT reached the maximum level at week 16, whereas the development of steatohepatitis began at week 12 (Xu, 2010). Treatment with I‐ol and I‐an significantly (*p* < 0.0167) diminished the increase in ALT activity by almost 70%, whereas that with UDCA exhibited a lesser effect. Studies on patients with NASH showed a non‐significant reduction of ALT by UDCA (Leuschner, 2010). Only I‐an in the highest dosage was able to reduce AST activity in a significant manner (*p* < 0.0167) but with a value of 90%.

Increased AP levels, as observed in the HFD group, are also reported frequently in patients with NASH (Hadizadeh, 2017). Only I‐an reduced the enhancement in enzyme activity almost completely in a very significant manner (*p* < 0.00333).

The serum level of γ‐GT in the HFD‐fed animals was significantly (*p* < 0.0167) elevated and all three compounds could reduce it to over 75% in a very highly significant manner (*p* < 0.000333). γ‐GT is an early predictive marker for IR, NAFLD, metabolic syndrome, and many other human diseases (Koenig, 2015). In particular, γ‐GT is considered to play an important role in the defense mechanism against oxidative stress by participating in the extracellular transport and cellular uptake of GSH (Lieberman, 1995). Thus, the animals in the HFD group may be exposed to massive oxidative stress.

Adiponectin and TNF‐α play a crucial role in the development of NASH (Tsochatzis, 2009). Rats in the HFD group showed reduced adiponectin levels associated with IR (Lu et al., 1997) as well as increased serum levels of TNF‐α, indicating NASH. Adiponectin on one hand and TNF‐alpha and leptin on the other influence each other in an antagonistic manner. The concentration of adiponectin was reduced in the serum of rats in the HFD group, though they did not gain weight and their insulin and glucose levels had not, or hardly, increased. Thus, the animals showed no sign of being overweight or manifesting IR or even type 2 diabetes.

In the case of ASH, a reduced adiponectin concentration was reported to be primarily related to the oxidative stress caused by CYP2E1 (Tang, 2012). In addition, the effect of alcohol on fatty tissue has been well‐documented. However, there are very few reports associating NASH with the role of CYP2E1 in adipose tissue. A study on a leptin‐deficient mouse model of diabetes mellitus concluded that the concentration of adiponectin decreased during the transition of non‐alcoholic fatty liver to NASH, whereas it remained constant in the absence of steatosis (Handa, 2014). However, the degree of steatosis was correlated with the body weight of the animals. I‐ol restored the serum level of adiponectin to that in the control group rats in a very significant manner (*p* < 0.00333); however, I‐an and UDCA significantly (*p* < 0.0167) exhibited slightly lesser effects.

A higher level of TNF‐α has been reported in patients with NAFLD and NASH by several studies; however, this marker does not allow the staging of NAFLD (Hadizadeh, 2017). In particular, I‐ol and UDCA significantly (*p* < 0.0167) reduced the elevated TNF‐α levels, whereas I‐an showed no effect.

The serum concentration of leptin in the HFD‐fed rats increased permanently from week 1–16 compared to that in rats fed the standard diet (Gauthier, 2006). However, a disagreement exists about whether the same is true for patients with NASH (Tsochatzis, 2009). In this study, the rats in the disease group showed a strong but not statistically significant increase in leptin level compared to that in the control group. I‐ol and I‐an, at their highest concentrations, significantly (*p* < 0.0167) reduced the effects of HFD to almost to the initial level in the control group.

All animals in the HFD group showed only a partial change in the lipid parameters of the liver and serum compared to those in the control group. In particular, the amount of triglycerides in the liver was increased in a very highly significant manner (*p* < 0.000333) in accordance with the results observed in patients with NAFLD (41). This effect was reduced by all three test compounds in a significant (*p* < 0.0167) to highly significant (*p* < 0.00333) manner. This clarity both in the efficacy of the drug candidates and in the level of disease value did not apply to the other lipid parameters, such as hepatic cholesterol and phospholipids as well as VLDL, LDL, and HDL cholesterol in the serum.

In contrast with other study results (Puri, 2007), we found the amount of cholesterol in the liver of the animals in the HFD group to be only slightly and non‐significantly increased compared to that in the control groups. In another study on patients with histologically confirmed NASH, a correlation was established between the severity of NAFLD and the amount of hepatic cholesterol (Min, 2012). The driving force was assumed to be the dysregulation of HMG‐CoA reductase and the associated accumulation of free cholesterol, which was not measured in our current study. The discrepancy in these results could be due to the export of hepatic cholesterol from the liver. Furthermore, disease progression in the HFD group may have not reached the stage where cholesterol accumulates in the liver or our animal model may not be suitable in this regard for comparing our results with those in patients with NASH. Moreover, the administration of a cholesterol‐free diet in our animal model and the composition of the liquid diet may also need to be considered. UDCA increases hepatic cholesterol level through increased synthesis of cholesterol in patients with pathological obesity (Mueller, 2015). This observation could explain the lack of an effect of UDCA in our study, whereas both the drug candidates were able to reduce the level of hepatic cholesterol below that in the control groups, but in a non‐statistically significant way.

In our study, the serum LDL level in the disease group was only slightly and non‐significantly increased compared to that in the control groups, whereas the HDL level showed a statistically significant (*p* < 0.0167) decrease. A decrease in the HDL levels was observed in obese patients with NAFLD (Min, 2012) and, in principle, in patients with NASH (Fujita, 2009). I‐ol was able to significantly (*p* < 0.0167) lower the LDL levels, whereas neither our drug candidates nor UDCA could elicit any effect on the HDL levels. The serum VLDL level is significantly higher in patients with steatosis or NASH (Fujita, 2009). The animals in our disease group had non‐significantly increased serum VLDL levels; I‐ol, I‐an, and UDCA appeared to reduce the release of VLDL from the liver.

In line with the lack of increase in body and liver weight in the disease group, these parameters—with the exception of hepatic triglycerides—could not clearly reflect the disease severity of the animals in the HFD group and the therapeutic success of the drug candidates. The contradicting results of our lipid parameters are comparable to some results reported in scientific publications. There was no increase in serum triglycerides (Xu, 2010) and no gain in body weight (Lieber et al., 2004) in the HFD group compared to that in the control group. This observation was contradicted by results from other animal experiments in which solid food was used and an increase in liver weight was recorded weeks before an increase in body weight (Xu, 2010). In our study, the lack of an increase in the body weight may have been due to the basic components of the liquid diet. HPMC or guar gum had previously caused reduced weight gain in rats administered an HFD (Brockman, 2014). Comparable results have been reported for mice and hamsters as well (Ban et al., 2012; Kim et al., 2015).

Considering that NASH can also induce fibrosis, we determined the amount of connective tissue in liver sections. The liver of the HFD‐fed animals showed a significant (*p* < 0.0167) to highly significant (*p* < 0.00333) increase in connective tissue compared to that in both the control groups, suggesting an induction of fibrosis. This observation had been described in the literature, although fibrosis has been found to be induced only after week 24 (Xu, 2010). Of the two drug candidates, I‐ol could possibly exert an antifibrotic effect.

The results of our histopathological examinations of the liver were consistent with observations from other experiments (Wang et al., 2008) and found to be comparable with those reported in human patients in the final stage of NASH (Takahashi & Fukusato, 2014): a pronounced macrovesicular steatosis, massive necrotic areas, and infiltrations by macrophages and lymphocytes in the disease group. The animals were considered to have “probably NASH,” according to the criteria of the NAFLD activity score for clinical studies published by Kleiner DE et al. (Kleiner et al., 2005). This was supported by our semi‐quantitative analysis and the increased area of connective tissue in the liver of HFD‐fed animals, which might add at least 1 point to the activity scores. Treatment with the test compounds decreased this value to 3 or even lower. Hence, the tissues were diagnosed as ‘not‐NASH’. These results were underlined by a dose‐dependent reduction of the pathological signs, especially by I‐ol: At first, steatotic vesicles and, finally, infiltration of mononuclear cells disappeared with increasing concentrations of I‐ol. UDCA showed no effect.

All the measured parameters (levels of SRA and hydrogen peroxide, equivalents of lipid peroxidation, and reduced glutathione level) following ROS stress in the rat livers of the disease group changed quite significantly compared to that in the control groups—with the exception of catalase activity. Glutathione level was reduced so drastically that an exhaustion of the cellular protective system against ROS had to be assumed. Thus, the administration of an HFD caused a severe burden on the liver owing to ROS stress. Both the drug candidates were able to relieve ROS stress in a significant (*p* < 0.0167) to very highly significant (*p* < 0.000333) and partially dose‐dependent manner. However, only I‐an affected GSH activity but not that of GR. UDCA had no effect on hydrogen peroxide level and reduced GSH activity. This observation was consistent with the results of a study that denied the ROS‐protective effect of UDCA in patients with NAFLD (Mueller, 2017).

The importance of CYP2E1 as an essential pathological source of intracellular ROS has been widely recognized in the published literature. The animal model used by us showed a significant increase in the mRNA and protein levels of CYP2E1 in the rat liver (Abdelmegeed, 2012; Lieber et al., 2004). Increased enzymatic activity of CYP2E1 in patients with NASH had also been reported previously (Aljomah et al., 2015; Baker, 2010; Chalasani et al., 2003; Weltman et al., 1998), whereas no difference in the mRNA levels was observed between patients with steatosis and NASH (Aljomah et al., 2015). We have shown, through in vitro experiments, that I‐ol and I‐an are strong inhibitors of CYP2E1 and able to counteract ROS‐induced stress (Diesinger et al., 2020). Overall, the results suggested that lots of the ROS‐induced stress in the HFD‐fed animals in our experiments may be due to the CYP2E1 enzyme activity (Lieber et al., 2004). However, further experiments must follow to prove the causal link between ROS stress caused by CYP2E1 overexpression and the development of NFALD.

In the acute toxicity study on rats, conducted according to the ICH guidelines, no acute effect was observed up to a dose of 500 g/kg b.w. for both drug candidates. However, two of the five rats that were administered I‐an eventually died, whereas I‐ol did not lead to any death. The LD_50_ value was estimated to be beyond 1000 mg/kg b.w. for both drug candidates. These observations could be correlated with the binding strength of the inhibitors to CYP2E1, which indicated that I‐ol exhibited the highest affinity, followed by I‐an (Diesinger et al., 2020). Overall, however, the acute toxic effect of both substances was so low that no classification system, according to the usual criteria, could be used (Walum, 1998).

## 5 CONCLUSIONS

The pathological parameters and histological staining data indicated that the Wistar rats of the HFD‐disease group developed NAFLD, in general, and NASH, in particular; however, the surrogate markers could not distinguish between steatosis and NASH. Both drug candidates influenced the degree of NASH, partly in a dose‐dependent manner, and showed an overall superior effectiveness over UDCA. This concerned in particular the ROS status in the liver, which was presumably generated in large part by the increased enzymatic activity of CYP2E1 in the course of the disease. They were shown to lack acute toxicity. As competitive inhibitors at the active site of CYP2E1, the ω‐imidazolyl‐alkyl derivatives I‐ol and I‐an could be a new rational therapeutic approach of NAFLD and especially of NASH.

Nevertheless, it was not possible to clarify what was the reason for the partially not equal effects of the two drug candidates. It also remained unclear why a linear dose–response relationship did not exist throughout the study. It should be remembered that not every laboratory parameter has the same clinical significance. There are also different correlations between the laboratory parameters, ROS and CYP2E1. Further analyses must follow to understand the rationality of CYP2E1 inhibition in NAFLD therapy. Although we demonstrated the low acute toxicity of the drug candidates, the potential toxic side effects of chronic oral application were not evaluated in this study. In particular, off‐target effects in the domain of drug‐metabolizing CYP‐450 isoenzymes should be explored in future. Furthermore, the therapeutic activity of these inhibitors should be evaluated in a different animal model for relevance to human NASH, such as a model based on the so‐called Western diet. Additionally, we need to explore the feasibility of other methods for the continuous diagnosis and observation of the animals, for example, MRT for small animals.

## CONFLICTS OF INTEREST

There are no conflicts of interest.

6

## References

[phy214795-bib-0001] Abdelmegeed, M. A. , Banerjee, A. , Yoo, S.‐H. , Jang, S. , Gonzalez, F. J. , & Song, B.‐J. (2012). Critical role of cytochrome P450 2E1 (CYP2E1) in the development of high fat‐induced nonalcoholic steatohepatitis. Journal of Hepatology, 57(4), 860–866. 10.1016/j.jhep.2012.05.019. 22668639 PMC3445664

[phy214795-bib-0002] Ahmed, M. (2015). Non‐alcoholic fatty liver disease in 2015. World Journal of Hepatology, 7, 1450–1459. 26085906 10.4254/wjh.v7.i11.1450 PMC4462685

[phy214795-bib-0003] Aljomah, G. , Baker, S. S. , Liu, W. , Kozielski, R. , Oluwole, J. , Lupu, B. , Baker, R. D. , & Zhu, L. (2015). Induction of CYP2E1 in non‐alcoholic fatty liver diseases. Experimental and Molecular Pathology, 99, 677–681. 26551085 10.1016/j.yexmp.2015.11.008 PMC4679539

[phy214795-bib-0004] Baker, S. S. , Baker, R. D. , Liu, W. , Nowak, N. J. , & Zhu, L. (2010). Role of alcohol metabolism in non‐alcoholic steatohepatitis. PLoS One, 5(3), e9570. 10.1371/journal.pone.0009570. 20221393 PMC2833196

[phy214795-bib-0005] Ban, S. J. , Rico, C. W. , Um, I. C. , & Kang, M. Y. (2012). Antihyperglycemic and antioxidative effects of Hydroxyethyl Methylcellulose (HEMC) and Hydroxypropyl Methylcellulose (HPMC) in mice fed with a high fat diet. International Journal of Molecular Sciences, 13, 3738–3750. 22489179 10.3390/ijms13033738 PMC3317739

[phy214795-bib-0006] Beers, R. F. Jr & Sizer, I. W. (1952). A spectrophotometric method for measuring the breakdown of hydrogen peroxide by catalase. Journal of Biological Chemistry, 195, 133–140. 14938361

[phy214795-bib-0007] Bessone, F. , Razori, M. V. , & Roma, M. G. (2019). Molecular pathways of nonalcoholic fatty liver disease development and progression. Cellular and Molecular Life Sciences, 76, 99–128. 30343320 10.1007/s00018-018-2947-0 PMC11105781

[phy214795-bib-0008] Bhagat, V. , Mindikoglu, A. L. , Nudo, C. G. , Schiff, E. R. , Tzakis, A. , & Regev, A. (2009). Outcomes of liver transplantation in patients with cirrhosis due to nonalcoholic steatohepatitis versus patients with cirrhosis due to alcoholic liver disease. Liver Transplantation, 15, 1814–1820. 19938128 10.1002/lt.21927

[phy214795-bib-0009] Brockman, D. A. , Chen, X. , & Gallaher, D. D. (2014). High‐viscosity dietary fibers reduce adiposity and decrease hepatic steatosis in rats fed a high‐fat diet. Journal of Nutrition, 144(9), 1415–1422. 10.3945/jn.114.191577. 24991042

[phy214795-bib-0010] Buege, J. A. , & Aust, S. D. (1978). Microsomal lipid peroxidation. Methods in Enzymology, 52, 302–310. 672633 10.1016/s0076-6879(78)52032-6

[phy214795-bib-0011] Buko, V. U. , Lukivskaya, O. Y. , Zavodnik, L. V. , Sadovnichy, V. V. , Petushok, N. E. , & Tauschel, N. D. (2002). Antioxidative effect of ursodeoxycholic acid in the liver of rats with oxidative stress caused by gamma‐irradiation. Ukrainskii Biokhimicheskii Zhurnal, 1999(74), 88–92. 12199106

[phy214795-bib-0012] Burstein, M. , & Scholnick, H. R. (1973). Turbidimetric estimation of chylomicrons and very low density lipoproteins in human sera after precipitation by sodium lauryl sulfate. Biomedicine, 19, 16–19. 4351005

[phy214795-bib-0013] Chalasani, N. , Gorski, J. C. , Asghar, M. S. , Asghar, A. , Foresman, B. , Hall, S. D. , & Crabb, D. W. (2003). Hepatic cytochrome P450 2E1 activity in nondiabetic patients with nonalcoholic steatohepatitis. Hepatology, 37, 544–550. 12601351 10.1053/jhep.2003.50095

[phy214795-bib-0014] Diesinger, T. , Buko, V. , Lautwein, A. , Dvorsky, R. , Belonovskaya, E. , Lukivskaya, O. , Naruta, E. , Kirko, S. , Andreev, V. , Buckert, D. , Bergler, S. , Renz, C. , Schneider, E. , Kuchenbauer, F. , Kumar, M. , Gunes, C. , Buchele, B. , Simmet, T. , Muller‐Enoch, D. , … Haehner, T. (2020). Drug targeting CYP2E1 for the treatment of early‐stage alcoholic steatohepatitis. PLoS One, 15, e0235990. 32701948 10.1371/journal.pone.0235990 PMC7377376

[phy214795-bib-0015] Drescher, H. K. , Weiskirchen, S. , & Weiskirchen, R. (2019). Current Status in Testing for Nonalcoholic Fatty Liver Disease (NAFLD) and Nonalcoholic Steatohepatitis (NASH). Cells, 8. 10.3390/cells8080845 PMC6721710 31394730

[phy214795-bib-0016] Ellman, G. L. (1959). Tissue sulfhydryl groups. Archives of Biochemistry and Biophysics, 82, 70–77. 13650640 10.1016/0003-9861(59)90090-6

[phy214795-bib-0017] Engin, A. (2017). Non‐alcoholic fatty liver disease. Advances in Experimental Medicine and Biology, 960, 443–467. 28585211 10.1007/978-3-319-48382-5_19

[phy214795-bib-0018] Findlay, J. B. C. E. , & Evans, W. H. E. (1987). Biological membranes: A practical approach. IRL Press.

[phy214795-bib-0019] Fujita, K. , Nozaki, Y. , Wada, K. , Yoneda, M. , Fujimoto, Y. , Fujitake, M. , Endo, H. , Takahashi, H. , Inamori, M. , Kobayashi, N. , Kirikoshi, H. , Kubota, K. , Saito, S. , & Nakajima, A. (2009). Dysfunctional very‐lowdensity lipoprotein synthesis and release is a key factor in nonalcoholic steatohepatitis pathogenesis. Hepatology. 10.1002/hep.23094 19650159

[phy214795-bib-0020] Gauthier, M. S. , Favier, R. , & Lavoie, J.‐M. (2006). Time course of the development of non‐alcoholic hepatic steatosis in response to high‐fat diet‐induced obesity in rats. British Journal of Nutrition, 95(2), 273–281. 10.1079/BJN20051635. 16469142

[phy214795-bib-0021] Hadizadeh, F. , Faghihimani, E. , & Adibi, P. (2017). Nonalcoholic fatty liver disease: Diagnostic biomarkers. World Journal of Gastrointestinal Pathophysiology, 8(2), 11– 10.4291/wjgp.v8.i2.11. 28573064 PMC5437499

[phy214795-bib-0022] Handa, P. , Maliken, B. D. , Nelson, J. E. , Morgan‐Stevenson, V. , Messner, D. J. , Dhillon, B. K. , Klintworth, H. M. , Beauchamp, M. , Yeh, M. M. , Elfers, C. T. , Roth, C. L. , & Kowdley, K. V. (2014). Reduced Adiponectin Signaling Due to Weight Gain Results in Nonalcoholic Steatohepatitis Through Impaired Mitochondrial Biogenesis. Hepatology, 60(1), 133–145. 10.1002/hep.26946. 24464605 PMC5993561

[phy214795-bib-0023] Hirano, T. , Ito, Y. , Saegusa, H. , & Yoshino, G. (2003). A novel and simple method for quantification of small, dense LDL. Journal of Lipid Research, 44, 2193–2201. 12897184 10.1194/jlr.D300007-JLR200

[phy214795-bib-0024] Kim, H. R. , Lee, G. H. , Cho, E. Y. , Chae, S. W. , Ahn, T. , & Chae, H. J. (2009). Bax inhibitor 1 regulates ER‐stress‐induced ROS accumulation through the regulation of cytochrome P450 2E1. Journal of Cell Science, 122, 1126–1133. 19339548 10.1242/jcs.038430

[phy214795-bib-0025] Kim, H. , Wang, Q. , Shoemaker, C. F. , Zhong, F. , Bartley, G. E. , & Yokoyama, W. H. (2015). Polysaccharide gel coating of the leaves of Brasenia schreberi lowers plasma cholesterol in hamsters. J Tradit Complement Med, 5, 56–61. 26151010 10.1016/j.jtcme.2014.10.003 PMC4488095

[phy214795-bib-0026] Kleiner, D. E. , Brunt, E. M. , van Natta, M. , Behling, C. , Contos, M. J. , Cummings, O. W. , Ferrell, L. D. , Liu, Y. C. , Torbenson, M. S. , Unalp‐Arida, A. , Yeh, M. , McCullough, A. J. , Sanyal, A. J. , & Nonalcoholic steatohepatitis clinical research , (2005). Design and validation of a histological scoring system for nonalcoholic fatty liver disease. Hepatology, 41, 1313–1321. 15915461 10.1002/hep.20701

[phy214795-bib-0027] Koenig, G. , & Seneff, S. (2015). Gamma‐glutamyltransferase: A predictive biomarker of cellular antioxidant inadequacy and disease risk. Disease Markers, 2015, 1–18. 10.1155/2015/818570. PMC4620378 26543300

[phy214795-bib-0028] Koppe, S. W. , Sahai, A. , Malladi, P. , Whitington, P. F. , & Green, R. M. (2004). Pentoxifylline attenuates steatohepatitis induced by the methionine choline deficient diet. Journal of Hepatology, 41, 592–598. 15464239 10.1016/j.jhep.2004.06.030

[phy214795-bib-0029] Lee, G. H. , Oh, K. J. , Kim, H. R. , Han, H. S. , Lee, H. Y. , Park, K. G. , Nam, K. H. , Koo, S. H. , & Chae, H. J. (2016). Effect of BI‐1 on insulin resistance through regulation of CYP2E1. Scientific Reports, 6, 32229. 27576594 10.1038/srep32229 PMC5006057

[phy214795-bib-0030] Leuschner, U. F. H. , Lindenthal, B. , Herrmann, G. , Arnold, J. C. , Rössle, M. , Cordes, H.‐J. , Zeuzem, S. , Hein, J. , & Berg, T. (2010). High‐dose ursodeoxycholic acid therapy for nonalcoholic steatohepatitis: A double‐blind, randomized, placebo‐controlled trial. Hepatology, 52(2), 472–479. 10.1002/hep.23727. 20683947

[phy214795-bib-0031] Lieber, C. S. , Leo, M. A. , Mak, K. M. , Xu, Y. , Cao, Q. , Ren, C. , Ponomarenko, A. , & Decarli, L. M. (2004). Model of nonalcoholic steatohepatitis. American Journal of Clinical Nutrition, 79, 502–509. 10.1093/ajcn/79.3.502 14985228

[phy214795-bib-0032] Lieberman, M. W. , Barrios, R. , Carter, B. Z. , Habib, G. M. , Lebovitz, R. M. , Rajagopalan, S. , Sepulveda, A. R. , Shi, Z. Z. , & Wan, D. F. (1995). y‐GIutamyl Transpeptidase. American Journal of Pathology, 147(5), 1175–1185. PMC1869519 7485380

[phy214795-bib-0033] Lu, P. , Alterman, M. A. , Chaurasia, C. S. , Bambal, R. B. , & Hanzlik, R. P. (1997). Heme‐coordinating analogs of lauric acid as inhibitors of fatty acid omega‐hydroxylation. Archives of Biochemistry and Biophysics, 337, 1–7. 8990261 10.1006/abbi.1996.9768

[phy214795-bib-0034] Ludwig, J. , Viggiano, T. R. , McGill, D. B. , & Oh, B. J. (1980). Nonalcoholic steatohepatitis: Mayo clinic experiences with a hitherto unnamed disease. Mayo Clinic Proceedings, 55, 434–438. 7382552

[phy214795-bib-0035] Min, H.‐K. , Kapoor, A. , Fuchs, M. , Mirshahi, F. , Zhou, H. , Maher, J. , Kellum, J. , Warnick, R. , Contos, M. J. , & Sanyal, A. J. (2012). Increased hepatic synthesis and dysregulation of cholesterol metabolism is associated with the severity of nonalcoholic fatty liver disease. Cell Metabolism, 15(5), 665–674. 22560219 10.1016/j.cmet.2012.04.004 PMC3361911

[phy214795-bib-0036] Mueller, M. , Castro, R. E. , Thorell, A. , Marschall, H.‐U. , Auer, N. , Herac, M. , Rodrigues, C. M. P. , & Trauner, M. (2017). Ursodeoxycholic acid: Effects on hepatic unfolded protein response, apoptosis and oxidative stress in morbidly obese patients. Liver International: Official Journal of the International Association for the Study of the Liver, 38(3), 523–531. 28853202 10.1111/liv.13562 PMC5836915

[phy214795-bib-0037] Mueller, M. , Thorell, A. , Claudel, T. , Jha, .P. , Koefeler, H. , Lackner, C. , Hoesel, B. , Fauler, G. , Stojakovic, T. , Einarsson, C. , Marschall, H.‐U. , & Trauner, M. (2015). Ursodeoxycholic acid exerts farnesoid X receptor‐antagonistic effects on bile acid and lipid metabolism in morbid obesity. Journal of Hepatology, 62(6), 1398–1404. 25617503 10.1016/j.jhep.2014.12.034 PMC4451470

[phy214795-bib-0038] Muller‐Peddinghaus, R. (1987). Pathophysiology and pharmacology of reactive oxygen species in inflammation. Arzneimittel‐Forschung, 37, 589–600. 3619980

[phy214795-bib-0039] Puri, P. , Baillie, R. A. , Wiest, M. M. , Mirshahi, F. , Choudhury, J. , Cheung, O. , Sargeant, C. , Contos, M. J. , & Sanyal, A. J. (2007). A lipidomic analysis of nonalcoholic fatty liver disease. Hepatology, 46(4), 1081–1090. 17654743 10.1002/hep.21763

[phy214795-bib-0040] Robertson, G. , Leclercq, I. , & Farrell, G. C. (2001). Nonalcoholic steatosis and steatohepatitis. II. Cytochrome P‐450 enzymes and oxidative stress. American Journal of Physiology. Gastrointestinal and Liver Physiology, 281, G1135–G1139. 11668021 10.1152/ajpgi.2001.281.5.G1135

[phy214795-bib-0041] Sharma, D. L. , Lakhani, H. V. , Klug, R. L. , Snoad, B. , El‐Hamdani, R. , Shapiro, J. I. , & Sodhi, K. (2017). Investigating molecular connections of non‐alcoholic fatty liver disease with associated pathological conditions in west virginia for biomarker analysis. J Clin Cell Immunol, 8, 10.4172/2155-9899.1000523. PMC5701750 29177105

[phy214795-bib-0042] Takahashi, Y. , & Fukusato, T. (2014). Histopathology of nonalcoholic fatty liver disease/nonalcoholic steatohepatitis. World Journal of Gastroenterology, 20, 15539–15548. 25400438 10.3748/wjg.v20.i42.15539 PMC4229519

[phy214795-bib-0043] Tang, H. , Sebastian, B. M. , Axhemi, A. , Chen, X. , Hillian, A. D. , Jacobsen, D. W. , & Nagy, L. E. (2012). Ethanol‐induced oxidative stress via the CYP2E1 pathway disrupts adiponectin secretion from adipocytes. Alcoholism: Clinical and Experimental Research, 36(2), 214–222. 10.1111/j.1530-0277.2011.01607.x. PMC3235233 21895711

[phy214795-bib-0044] Tsochatzis, E. A. , Papatheodoridis, G. V. , & Archimandritis, A. J. (2009). Adipokines in nonalcoholic steatohepatitis: From pathogenesis to implications in diagnosis and therapy. Mediators of Inflammation, 2009, 1–8. 10.1155/2009/831670. PMC2694309 19753129

[phy214795-bib-0045] Vernon, G. , Baranova, A. , & Younossi, Z. M. (2011). Systematic review: the epidemiology and natural history of non‐alcoholic fatty liver disease and non‐alcoholic steatohepatitis in adults. Alimentary Pharmacology & Therapeutics, 34, 274–285. 21623852 10.1111/j.1365-2036.2011.04724.x

[phy214795-bib-0046] Walum, E. (1998). Acute oral toxicity. Environmental Health Perspectives, 106(Suppl 2), 497–503. 9599698 10.1289/ehp.98106497 PMC1533392

[phy214795-bib-0047] Wang, Y. , Ausman, L. M. , Russell, R. M. , Greenberg, A. S. , & Wang, X. D. (2008). Increased apoptosis in high‐fat diet‐induced nonalcoholic steatohepatitis in rats is associated with c‐Jun NH2‐terminal kinase activation and elevated proapoptotic Bax. Journal of Nutrition, 138, 1866–1871. 10.1093/jn/138.10.1866 PMC2587062 18806094

[phy214795-bib-0048] Weltman, M. D. , Farrell, G. C. , Hall, P. , Ingelman‐Sundberg, M. , & Liddle, C. (1998). Hepatic cytochrome P450 2E1 is increased in patients with nonalcoholic steatohepatitis. Hepatology, 27, 128–133. 9425928 10.1002/hep.510270121

[phy214795-bib-0049] Xu, Z.‐J. , Fan, J.‐G. , Ding, X.‐D. , Qiao, L. , & Wan, G.‐L. (2010). Characterization of high‐fat, diet‐induced, non‐alcoholic steatohepatitis with fibrosis in rats. Digestive Diseases and Sciences, 55(4), 931–940. 19459046 10.1007/s10620-009-0815-3 PMC2946554

[phy214795-bib-0050] Yamaguchi, K. , Yang, L. , McCall, S. , Huang, J. , Yu, X. X. , Pandey, S. K. , Bhanot, S. , Monia, B. P. , Li, Y. X. , & Diehl, A. M. (2007). Inhibiting triglyceride synthesis improves hepatic steatosis but exacerbates liver damage and fibrosis in obese mice with nonalcoholic steatohepatitis. Hepatology, 45, 1366–1374. 17476695 10.1002/hep.21655

[phy214795-bib-0051] Younossi, Z. M. , Koenig, A. B. , Abdelatif, D. , Fazel, Y. , Henry, L. , & Wymer, M. (2016). Global epidemiology of nonalcoholic fatty liver disease‐Meta‐analytic assessment of prevalence, incidence, and outcomes. Hepatology, 64, 73–84. 26707365 10.1002/hep.28431

